# Genetic and clinical analysis of *TP73* gene in amyotrophic lateral sclerosis patients from Chinese mainland

**DOI:** 10.3389/fnagi.2023.1114022

**Published:** 2023-02-09

**Authors:** Xuxiong Tang, Yanchun Yuan, Zhen Liu, Yue Bu, Linxin Tang, Qianqian Zhao, Bin Jiao, Jifeng Guo, Lu Shen, Hong Jiang, Beisha Tang, Junling Wang

**Affiliations:** ^1^Department of Neurology, Xiangya Hospital, Central South University, Changsha, Hunan, China; ^2^National Clinical Research Center for Geriatric Diseases, Xiangya Hospital, Central South University, Changsha, Hunan, China; ^3^Key Laboratory of Hunan Province in Neurodegenerative Disorders, Central South University, Changsha, Hunan, China; ^4^Hunan International Scientific and Technological Cooperation Base of Neurodegenerative and Neurogenetic Diseases, Changsha, China; ^5^Engineering Research Center of Hunan Province in Cognitive Impairment Disorders, Central South University, Changsha, China; ^6^School of Basic Medical Science, Central South University, Changsha, Hunan, China

**Keywords:** amyotrophic lateral sclerosis, TP73, gene mutation, phenotype–genotype association, neurodegenerative disease, clinical characteristic

## Abstract

**Introduction:**

*TP73* was recently identified as a novel causative gene for amyotrophic lateral sclerosis (ALS). We aimed to determine the contribution of variations in *TP73* in the Chinese ALS population and to further explore the genotype-phenotype correlations.

**Methods:**

We screened rare, putative pathogenic *TP73* mutations in a large Chinese ALS cohort and performed association analysis of both rare and common *TP73* variations between cases and controls.

**Results:**

Of the 985 ALS patients studied, six rare, heterozygous putative pathogenic variants in *TP73* were identified among six unrelated sALS patients. Exon 14 of *TP73* might be a mutant hotspot in our cohort. Patients with ALS with only rare, putative pathogenic *TP73* mutations exhibited a characteristic clinical profile. Patients harboring multiple mutations in *TP73* and other ALS-related genes displayed a significantly earlier onset of ALS. Association analysis revealed that rare *TP73* variants in the untranslated regions (UTRs) were enriched among ALS patients; meanwhile, two common variants in the exon-intron boundary were discovered to be associated with ALS.

**Discussion:**

We demonstrate that *TP73* variations also have contributed to ALS in the Asian population and broaden the genotypic and phenotypic spectrum of *TP73* variants in the ALS-frontotemporal dementia (FTD) spectrum. Furthermore, our findings first suggest that *TP73* is not only a causative gene, but also exerts a disease-modifying effect. These results may contribute to a better understanding of the molecular mechanism of ALS.

## 1. Introduction

Amyotrophic lateral sclerosis (ALS) is a fatal neurodegenerative disorder characterized by the rapidly progressive loss of motor neurons in the brain and spinal cord, resulting in relentlessly worsening weakness and wasting of voluntary muscle until death from respiratory failure occurs, typically within 2–4 years of symptom onset (Al-Chalabi and Hardiman, 2013; Feldman et al., 2022; Goutman et al., 2022). ALS has generally been considered a relatively rare disease, nonetheless, the number of ALS patients worldwide is projected to reach 376,674 by 2040, predominantly due to aging (Al-Chalabi and Hardiman, 2013; Arthur et al., 2016). This rise is expected to put a huge socioeconomic strain on global healthcare systems. As with most neurodegenerative diseases, only 10% of ALS cases are hereditary, referred to as familial ALS (fALS), with the remaining 90% of cases classified as sporadic ALS (sALS) (Renton et al., 2014). ALS shows a significant degree of clinical and genetic heterogeneity, and still, much remains unclear about the etiology and pathophysiological mechanisms underlying this disease (Swinnen and Robberecht, 2014). Genetic variation contributed to ALS (Renton et al., 2014; Taylor et al., 2016). To date, over 40 genes have been identified as being implicated in the pathogenesis of ALS (Ghasemi and Brown, 2018).

Recently, a published study identified the gene of tumor protein P73 (*TP73*) as a novel causative gene for ALS (Russell et al., 2021). By screening the whole-exome sequencing (WES) data from a discovery cohort of 87 European patients with sALS and two replication cohorts comprising 2,853 individuals with sALS, the researchers discovered 24 rare protein-coding variants in *TP73*. To further validate the pathogenic role of *TP73* variants in ALS, they then performed functional experiments including C2C12 myoblast differentiation assays *in vitro* and spinal motor neuron (SMN) axonal branching assay *in vivo*. Furthermore, they first proposed that apoptosis in motor neurons may be involved in the pathology of ALS. Following that, Pereira and colleagues linked rare mutations in *TP73* with frontotemporal dementia (FTD), particularly primary progressive aphasia, which further corroborated the contribution of *TP73* variations in the ALS-FTD spectrum (Tabuas-Pereira et al., 2022).

However, mutation analysis of the *TP73* gene conducted on other countries and Asian populations is insufficient. A plethora of studies has highlighted that the genetic epidemiology of ALS varies among different racial groups (Zou et al., 2017). Therefore, we performed a thorough genetic analysis using whole-exome sequencing to investigate the contributions of *TP73* variations in the Chinese ALS population and further characterized the clinical features of these patients to explore the genotype–phenotype correlations in this work.

## 2. Materials and methods

### 2.1. Participants

In this study, a large cohort of 1,004 patients with ALS was enrolled from Xiangya Hospital, Central South University, in either an inpatient or outpatient setting. Each patient got a thorough examination and was diagnosed with ALS by at least two experienced neurologists based on the current Gold Coast criteria (Shefner et al., 2020). Detailed demographic data and clinical information were collected when enrolled and updated at follow-up visits. Participants having known pathogenic mutations in the established ALS causative genes were excluded first. In total, 1,258 neurological disease-free individuals of Chinese ancestry matched by geography were recruited as healthy controls (HCs). This study was approved by the Ethics Committee and the Expert Committee of Xiangya Hospital, Central South University. All participants completed written informed consent in compliance with the Helsinki Declaration.

### 2.2. WES analysis

Genomic DNA was prepared from peripheral blood leukocytes of each subject *via* standard extraction procedures. The purity and quantity of extracted DNA were detected with a NanoDrop spectrophotometer 1,000 (Thermo Scientific). WES was performed on all participants using a previously published method (Zeng et al., 2019). The resulting data were mapped to the reference genome (GRCh37/hg19). Variants with a depth of coverage <10, allele balance <0.25, or Phred quality score <20 were removed. Proceed to annotate variations with the ANNOVAR software (Wang et al., 2010). After the quality control, totaling 985 patients and 1,224 healthy controls were included for further analysis. The data generation processes have already been described in detail (Liu et al., 2021).

We screened for rare, putative pathogenic variants of *TP73* in the Chinese ALS population. Inclusion criteria for the rare, putative pathogenic variants were: (1). the frequency was lower than 0.001 in our in-house and the following public database: the 1,000 Genome Project-East Asian (1000G_EAS), the NHLBI Exome Sequencing Project-East Asian (ESP6500s_EAS), the Exome Aggregation Consortium-East Asian (ExAC_EAS), and the Genome Aggregation Database-East Asian (GnomAD_EAS); (2). absent from HCs (3). located in the protein-coding regions; (4). present in a heterozygous state; (5). annotated as missense, stop gain/loss, frameshift, or splice-site variants; and (6). rare missense variants predicted to be pathogenic by at least five of 11 *in silico* (Quadri et al., 2018). It’s worth emphasizing that, considering the relatively small number of our control group, we only judged the pathogenicity of a rare variant to be robust if it was absent from HCs.

### 2.3. The single common variant association test

The common and rare variants were defined according to the minor allele frequency (MAF) in all participants in our cohort (common variants: MAF ≥ 0.01; rare variants: MAF < 0.01).

To evaluate the association of *TP73* variants with ALS risk in the Chinese population, we conducted the single common variant association test between the ALS and control groups using the Mixed Linear Model (MLM). The extracted top three principal components of population stratification in the principal component analysis (PCA) were included as covariates in our cohort. The possible effect of common variants inside exon-intron boundaries on the *TP73* gene expression was assessed by Expression Quantitative Trait Locus (eQTL) analysis (Zhu et al., 2016), which was widely utilized to compare the gene expression levels among individuals with different genotypes. All data were available from the Genotype-Tissue Expression (GTEx) database.

### 2.4. The gene-based rare variants association test

For the gene-based rare variants association test, the cumulative burden of rare variants across defined genomic regions of *TP73* between cases and controls was evaluated using the optimal sequence kernel association test (SKAT-O) implemented in the R package SKAT (Lee et al., 2012). The SKAT-O test maximized the test power of detecting the target gene by unifying the advantages of both the burden test and SKAT (Lee et al., 2012). Gender, age, and WES coverage were considered as possible covariates for adjustment before computing the *p* value.

### 2.5. Statistical analysis

Descriptive statistics are reported as mean ± SD or median ± SD (standard deviation, SD) for continuous variables and percentages for categorical variables. The comparison of continuous variables was assessed by Student’s t-test. For categorical variables, the Fisher’s exact test or Chi-square test was used to verify the significant differences between the two compared groups. Statistical analyses were carried out in SPSS (version 26.0) software. All tests were two-tailed, and significance was set at *p* < 0.05.

## 3. Results

### 3.1. Demographics

In all, 985 patients with ALS and 1,224 healthy controls were analyzed in our study. The 985 ALS patients were on average 54.2 years old at the time of onset, and 56.2 years old at the time of sampling; the geographically matched controls were older (mean age 68.47 years). 75.3% (740/985) of our ALS cohort had limb onset, while 19.39% (191/985) had bulbar onset. Detailed demographic information for all individuals is shown in Supplementary Table S1.

### 3.2. Rare, putative pathogenic mutation analysis

**Figure 1:**
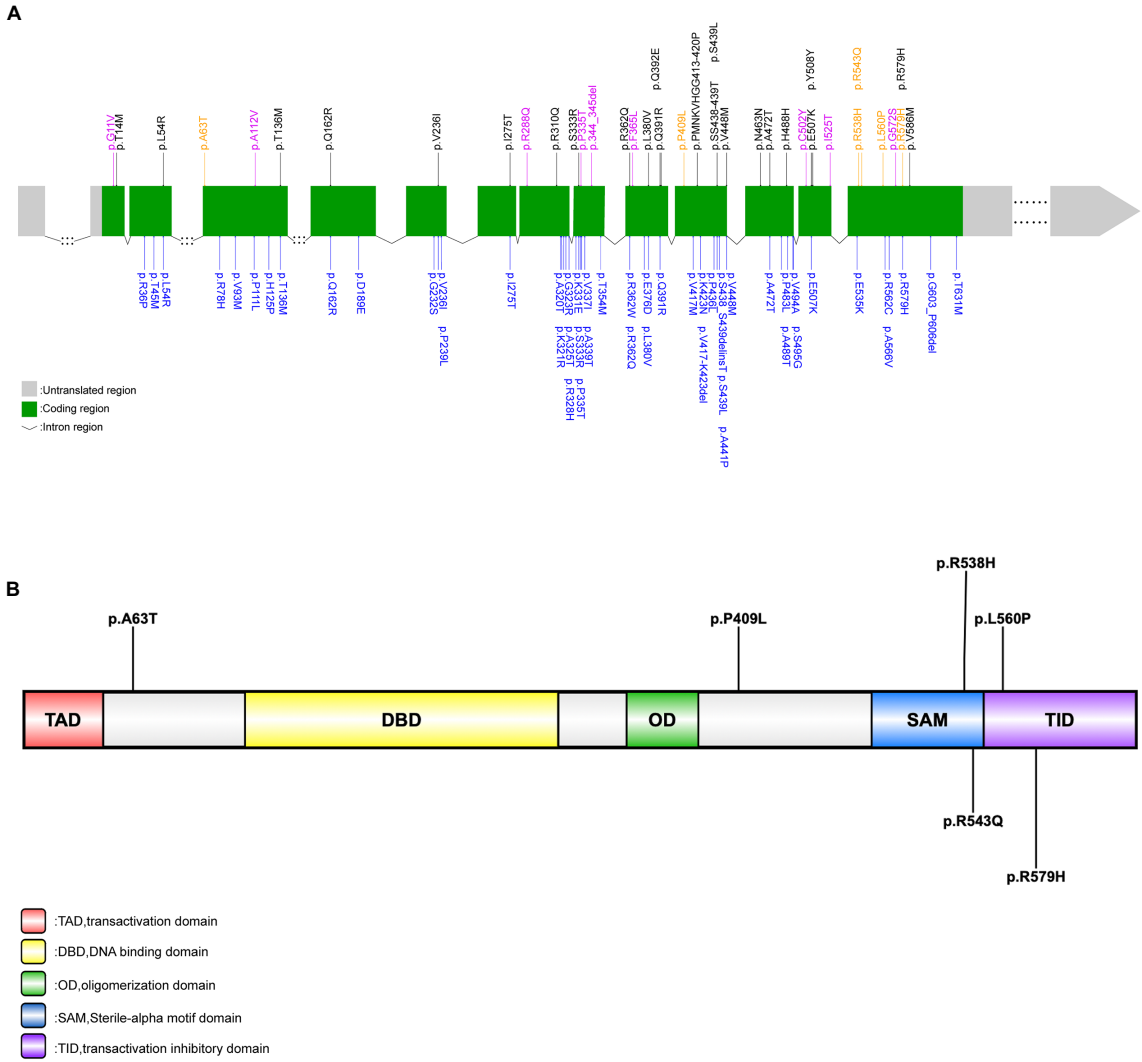
Rare *TP73* variants identified in Russell et al.’s (2021), Dilliott et al.’s (2022), and this study. **(A)** Schematic representation of the *TP73* transcript NM_005427. Rare, putative pathogenic missense variants identified in this study’s ALS cohort (yellow font); rare variants identified in this study’s control group (purple font); rare, protein-coding variants in *TP73* identified by Russell et al. (2021) (black font); rare, non-synonymous variants in *TP73* identified by Dilliott et al. (2022) (blue font). **(B)** Schematic representation of the TAp73 protein. Rare, putative pathogenic missense variants identified in this study (black font).

We screened all *TP73* exon regions and their surrounding sequences in each ALS patient. A total of six rare, heterozygous putative pathogenic mutations that fulfilled pathogenicity criteria were discovered among six unrelated sALS patients but not in HCs. The overall frequency of patients with rare pathogenic *TP73* mutations was 0.6%. All these six variations were protein-altering missense mutations. And the majority of them substituted amino acids that were highly conserved across species. The six variants were c.187G > A (p.A63T), c.1226C > T (p.P409L), c.1613G > A (p.R538H), c.1628G > A (p.R543Q), c.1679 T > C (p.L560P), and c.1736G > A (p.R579H). The variant, c.187G > A (p.A63T), was novel and absent from all databases. Three of these six variations (50%), namely c.187G > A (p.A63T), c.1226C > T (p.P409L), and c.1613G > A (p.R538H) have never been reported in association with the ALS-FTD spectrum. Interestingly, when investigating the distribution of these six potential pathogenic loci present in *TP73*, we discovered that a significant portion of them (4/6, 66.7%) were located inside exon 14, which differed from earlier studies. Consequently, we hypothesize that exon 14 of *TP73* might be a mutant hotspot in our ALS series. The location and pathogenicity information of these six variants identified in this study were summarized in Figure 1 and Supplementary Tables S2, S3 (Liu et al., 2015).

### 3.3. Clinical features of ALS patients with rare, putative pathogenic *TP73* mutations

For the six patients carrying potential pathogenic *TP73* variants described above, the age at disease onset ranged from 32 to 69 years old. We observed a completely masculine involvement since all six patients were men. In addition, we screened for other pathogenic mutations in the known ALS genes in these patients (Supplementary Table S4). There were no reported pathogenic mutations in the causative genes for ALS in each of these six individuals. Notably, two of the six patients (patient A0048 and patient S7918) had multiple variants in ALS-related genes with uncertain pathogenicity (patient A0048: *KIF5A* c.A86G: p.K29R and *TP73* c.1613G > A: p.R538H; patient S7918: *CCNF* c.2199delC: p.D733fs and *TP73* c.187G > A: p.A63T), and the remaining four patients only had *TP73* mutations. Considering the burden of multiple variants in ALS-causing genes on the disease expression, including the age at onset, progression rate, and survival, we separated these six patients into two subgroups for further clinical phenotype analysis (one subgroup with only *TP73* mutations, the other with multiple mutations in both *TP73* and other ALS-related genes) (Cady et al., 2015; Pang et al., 2017; Naruse et al., 2019). When the clinical phenotype analysis was restricted to patients with ALS with only *TP73* mutations, a distinct clinical profile emerges, with a considerably higher mean age at onset (61.5 ± 7.72 years), a shorter survival time (19.25 ± 10.01 months), and invariably limb onset. In the other subgroup, in contrast to patients carrying only *TP73* mutations, the age at onset in patients with ALS with multiple mutations is accelerated (patient A0048: 32-year-old, patient S7918:38-year-old). Furthermore, one of the six patients, S0423, suffered from cognitive impairment and behavioral problems and was eventually diagnosed with ALS-FTD. Most of these affected individuals exhibited typical symptoms and signs of the simultaneous impairment of upper and lower motor neurons. The clinical features of the six sALS patients were listed in Table 1.

**Table 1:** Clinical features of patients with ALS with rare, putative pathogenic variants in the *TP73* gene.

Patients no.	S0423	S3513	S4096	S5941	A0048	S7918
Variants	c.1226C > T (p.P409L)	c.1736G > A (p.R579H)	c.1628G > A (p.R543Q)	c.1679 T > C (p.L560P)	c.1613G > A (p.R538H)	c.187G > A (p.A63T)
Family history	S	S	S	S	S	S
Sex	M	M	M	M	M	M
Age at onset, y	65	61	69	51	32	38
Disease duration, m	13^a^	13^a^	17^a^	34	49	42
Site of onset	Spinal	Spinal	Spinal	Spinal	Spinal	Bulbar
Muscle weakness and atrophy	Generalized	Generalized	Generalized	lower limbs, right upper limb	Generalized	Lower limbs, right upper limb
Muscle fasciculation	NA	Extensive	Extensive	NA	Extensive	Right upper limb
Dysarthria	+	+	+	−	+	+
Dysphagia	+	+	+	−	+	+
Dyspnea	+	+	+	−	−	−
Autonomic dysfunction	−	−	−	+	−	−
Reflexes	Hyperreflexia	Hyperreflexia	Hyperreflexia	Normal	Hyperreflexia	Hyporeflexia in upper limbs; hyperreflexia in lower limbs
Cognition	FTD	Normal	Executive dysfunction and memory impairments	Normal	Executive dysfunction and memory impairments	Normal
Brain MRI	Brain atrophy	No obvious abnormalities	Brain atrophy	No obvious abnormalities	No obvious abnormalities	No obvious abnormalities
Education level, y	NA	0	9	8	9	15
MMSE score	#	20/30	NA	NA	NA	NA
ECAS score	#	NA	56/136	104/136	63/136	101/136
ALS-FRS score	32/48	31/48	41/48	43/48	26/48	35/48
EMG	Ongoing denervation and chronic reinnervation in all four segments	Ongoing denervation and chronic reinnervation in all four segments	Ongoing denervation and chronic reinnervation in all four segments	Ongoing denervation and chronic reinnervation in three segments	Ongoing denervation and chronic reinnervation in all four segments	Ongoing denervation and chronic reinnervation in all four segments

ALS, Amyotrophic lateral sclerosis; MMSE, Mini-Mental State Examination; ECAS, Edinburgh Cognitive and Behavioral ALS Screen; ALSFRS-R, ALS Functional Rating Scale–Revised; MRI, Magnetic resonance imaging; EMG, Electromyogram; NA, Data not available; +, Affected; −, Unaffected.

a The patient was dead at the time of the study.

#: Unable to complete the scale.

The variant, c.1226C > T (p.P409L), was identified in patient S0423, diagnosed with ALS-FTD. He was a farmer without specific environmental exposure. At the age of 65, he presented with muscle weakness in his upper limbs initiated from bilateral hands, which quickly extended to all four extremities in only 1 month. He then developed dysarthria and dysphagia 2 months later. Concurrently, his primary care providers complained of his language difficulties, behavioral changes, and cognitive impairment, which included trouble retrieving words, effortful speech restricted to short, simple sentences, hyperphagia, irritability, attacks on others, decreased memory, dropped comprehension, inability to find out things, and failure to understand some instructions. At the time of the first observation after 8 months of onset (at 66 years old), he was unable to walk or talk, and was reliant on family members for his daily requirements. On comprehensive neurological evaluation, he showed signs of upper motor neuron (UMN) damage and lower motor neuron (LMN) depletion features, such as positive palmomental reflex, hyperreflexia, and muscle atrophy of all four extremities. Electromyogram (EMG) revealed abundant and diffuse ongoing denervation (spontaneous potentials) and chronic reinnervation changes in all four segments (bulbar, cervical, thoracic, and lumbar). Brain magnetic resonance imaging (MRI) scan indicated mild age-related brain atrophy (Supplementary Figure S1). A battery of neuropsychological tests, including the Edinburgh Cognitive and Behavior ALS Screen (ECAS), the Mini-Mental State Examination (MMSE), the Montreal Cognitive Assessment (MoCA), and the Frontal Assessment Battery (FAB) was not completed due to his inability to communicate. He eventually died of respiratory failure 13 months after disease onset.

Another two male patients, S3513 and S4096, carrying variants c.1736G > A (p.R579H) and c.1628G > A (p.R543Q) separately, had similar clinical manifestations. They all began with muscle weakness in the right hand, at the ages of 61 and 69, respectively. They both developed muscle weakness and atrophy in all four limbs over the next year, along with extensive fasciculation, and dysarthria. Patient S3513 subsequently reported simultaneous involvement of the contralateral arm, and bilateral legs, accompanied by fasciculation, 5 months after disease onset. He developed dysarthria in the seventh month of onset, at which point the weakness of four limbs aggravated: he was unable to lift heavy objects with his upper limbs and had difficulty walking independently. For patient S4096 with a right-hand onset, his left upper limb and bulbar (choking) were reported as the second symptomatic sites. Following that, at the fifth month of the disease course, the weakness continuously progressed to both lower limbs, and dysarthria appeared. Their bilateral deep tendon reflexes were brisk, the Hoffman sign and palmomental reflex were present. EMGs both showed abundant and diffuse ongoing denervation as well as chronic reinnervation alterations at four segments. However, brain MRI revealed that patient S4096 with the c.1628G > A (p.R543Q) variant had mild brain atrophy (Supplementary Figure S1), whereas patient S3513 with the c.1736G > A (p.R579H) had no obvious abnormalities. ECAS score of patient S4096 was 56/136, and each subgroup score of this scale suggested that the decline was driven from executive dysfunction and memory impairments, rather than behavioral dysfunction. In the end, patient S3513 died at 13 months after onset and 17 months for patient S4096.

Another patient (S5941), carrying c.1679 T > C (p.L560P) variants, was a male, who complained of progressive muscle weakness and atrophy in his right arm at the 51-year-old. The weakness gradually extended to his bilateral lower limbs, making stair climbing difficult. Besides, he developed autonomic dysfunction, manifesting as sometimes excessive sweating during the day and heat intolerance, with no nocturnal sweating or cold intolerance, as revealed by the Scale for Outcomes in Parkinson’s disease (PD) for Autonomic Symptoms (SCOPA-AUT), a self-reported questionnaire widely used for the assessment of autonomic function in neurodegenerative disease (Visser et al., 2004; Damon-Perriere et al., 2012; Del et al., 2020; Assante et al., 2022). Neurological examination revealed no evidence of upper motor neuron damage, and EMG demonstrated abundant and diffuse ongoing denervation and chronic reinnervation changes in three segments (cervical, thoracic, and lumbar). No symptoms of dysphagia or dysarthria were found at the most recent follow-up visit after 34 months of onset. Based on all the available information thus far, he was clinically diagnosed with progressive muscular atrophy (PMA).

The remaining two patients, both harbored multiple variants in *TP73* and other ALS-related genes. Patient A0048 who carried the c.1613G > A (p.R538H) variant, and patient S7918 who had the c.187G > A (p.A63T) variant, were both young men with no family history of the condition, starting with ALS phenotype at 32 and 38-year-old, respectively. Patient A0048 first displayed weakness in his left lower extremity, and then spread fast in the early disease course. By the fourth month from symptom onward, he had weakness in all four limbs; he then gradually experienced bulbar muscle weakness, presenting with dysarthria, dysphagia, and bucking during the next 2 years. Patient S7918 showed bulbar onset, initially with dysarthria and dysphagia, then developed asymmetric weakness and atrophy in bilateral lower limbs and the right arm within 3 years, accompanied by extensive fasciculation. Neurological evaluation and EMGs on them both indicated evident signs of involvement of both upper and lower motor neurons. According to ECAS, patient A0048 had executive function deficits and memory impairments (total score: 63/136, subscore of executive function:14/60; subscore of memory: 3/24), whereas patient S7918 had no cognitive changes.

### 3.4. Association analysis and eQTL analysis of single common variants in *TP73*

**Figure 2:**
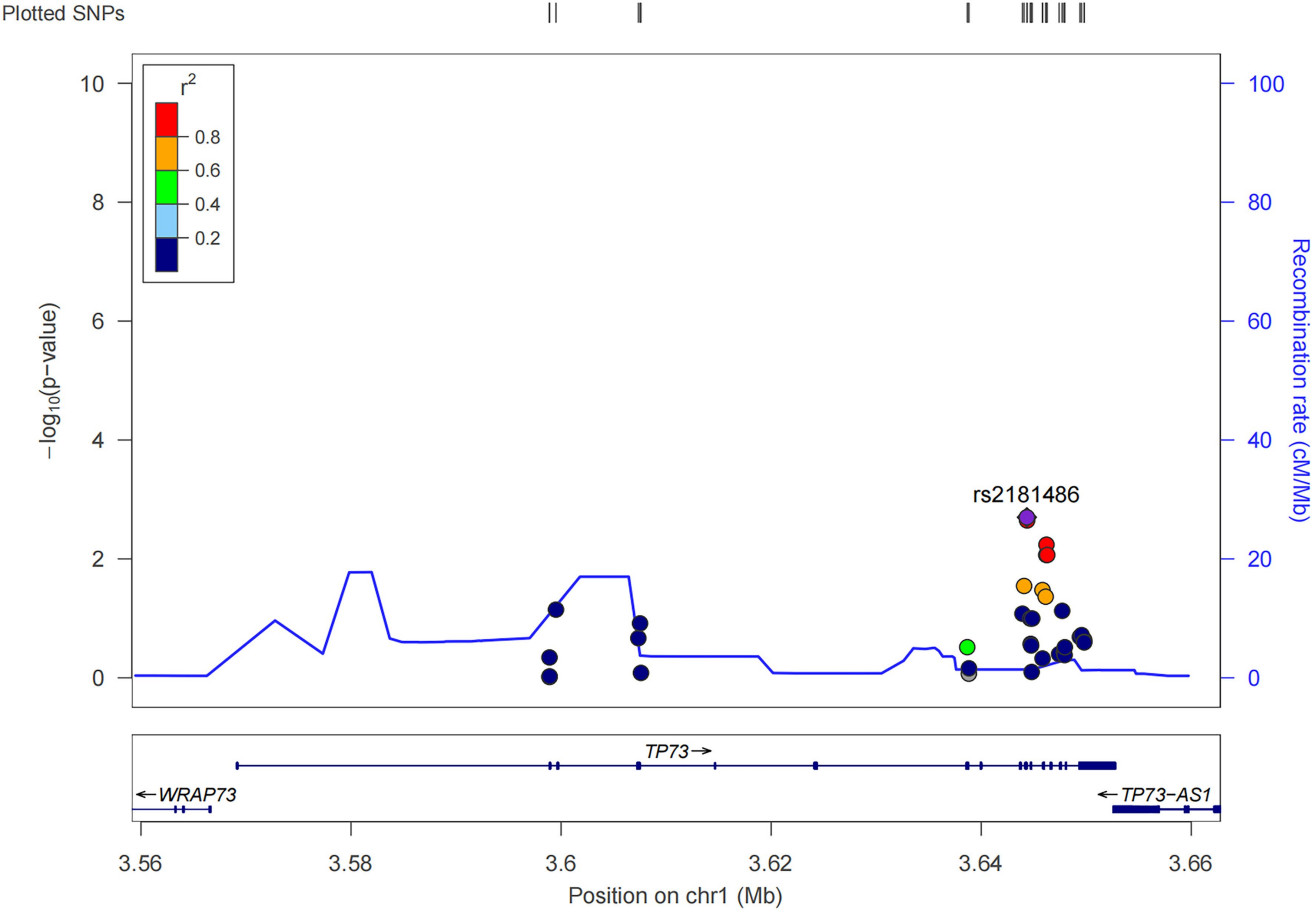
Locuszoom of the 36 common *TP73* variants identified in this study (http://locuszoom.org/).

We performed the single-variant analysis of each common variant discovered in the exon and near exon-intron boundary regions to ascertain the association between common variations of *TP73* and ALS. As shown in Figure 2, 36 common *TP73* variants were identified in our cohort (Figure 2, Supplementary Table S5). Then, utilizing PLINK 1.90, we obtained linkage disequilibrium (LD) statistics for these 36 common variants to plot linkage blocks (Supplementary Table S6). As 24 of the 36 common variants were mapped to 6 separate blocks, the estimated number of independent tests was 18, and the corresponding assumed Bonferroni-corrected significance threshold of *p* was 0.0028 (0.05/18). The resulting data revealed that none of the common variants inside the exon regions displayed significant univariate association with ALS. While two common variants rs2181486 and rs2146657 in the same block at exon-intron boundary, attained Bonferroni-corrected statistical significance (Table 2). Taking into consideration these two common variants in nearly perfect LD (the *R*^2^ of these two variants is 0.9850), we next chose the rs2181486 mutant locus to search the publicly accessible GTEx database to find if these two common variations impact the expression of TP73. Of note, based on the calculation of the eQTL algorithm, the rs2181486 variants might give rise to a decreased TP73 protein level in the cerebral cortex, spinal cord, as well as skeletal muscles (Table 3). These results provided suggestive evidence for association of ALS and common *TP73* variants in the near-exon intron boundaries.

**Table 2:** Association analysis for the two common variants achieving statistical significance in the *TP73* gene.

Position	Ref	Alt	dbSNP	Frequency	*N*	MAF_ case	MAF_ control	*p*-value	p^*^-value	SE
Chr1:3644349	A	G	rs2181486	0.3014	2,209	0.2766	0.3215	0.002	0.0359	0.0168
Chr1:3644374	A	G	rs2146657	0.2997	2,209	0.2751	0.3194	0.002	0.0406	0.0169

Chr: chromosome; Ref: reference allele; Alt: alternate allele; dbSNP: dbSNP137 (https://www.ncbi.nlm.nih.gov/snp/); *N*: number of subjects in this study; p^*^-value: *p*-value after the Bonferroni correction; SE: standard error.

**Table 3:** Effect of the common variant rs2181486 on TP73 protein expression in different regions of normal human brain and skeletal muscles.

rsID	Chr:position	Alt	Frequency of the Alt_cases	Frequency of the Alt_controls	Ensebl Gencode ID	Tissue	NES
rs2181486	chr1_3,644,349_A_G	G	0.2732	0.3213	ENSG00000078900.14	Brain - Cortex	0.24
rs2181486	chr1_3,644,349_A_G	G	0.2732	0.3213	ENSG00000078900.14	Brain - Spinal cord (cervical c-1)	0.13
rs2181486	chr1_3,644,349_A_G	G	0.2732	0.3213	ENSG00000078900.14	Muscle - Skeletal	0.24

Chr: chromosome; Alt: alternate allele; NES: normalized effect size.

### 3.5. Burden analysis of rare *TP73* variants at the gene level

We applied the SKAT-O test to compare the aggregate burden of rare variants residing in the coding regions, untranslated regions, and intron-exon boundaries of *TP73* between ALS cases and HCs, separately. Compared with HCs, the frequency of carriers of rare *TP73* variants in the coding regions and intron-exon boundaries was not significantly higher in the ALS group. We observed an enrichment of rare *TP73* variations in the UTRs among ALS cases, with a *p* value of 0.046 for the cumulative burden of rare *TP73* variations in the UTRs between the two compared groups, indicating that rare *TP73* UTRs variants were significantly associated with ALS (Supplementary Table S7).

## 4. Discussion

The p73 protein belonging to the p53 family, encoded by the *TP73* gene, has five functional domains: a transactivation domain (TAD), a DNA binding domain (DBD), an oligomerization domain (OD), a Sterile-alpha motif domain (SAM), and a transactivation inhibitory domain (TID) (Figure 1B; Jost et al., 1997; Dotsch et al., 2010; Osterburg and Dotsch, 2022). The p73 is a multifunctional protein in neurobiology and P73-deficient mice were found to develop neurological defects (Pozniak et al., 2000; Yang et al., 2000; Lee et al., 2004). Wetzel et al. (2008) demonstrated that p73 haploinsufficiency can cause age-related neuronal degeneration, indicating that p73 is implicated in neurodegeneration diseases. Recently, a large case–control research demonstrated *TP73* as a new ALS risk gene, although a replication study yielded conflicting results (Dilliott et al., 2022). Of note, neither of these two studies included the Asian population. Considering the distinct genetic architecture among different ethnicities, we screened *TP73* variants in a large Chinese ALS cohort to evaluate the contribution of *TP73* variants to Chinese ALS patients.

We identified six rare, heterozygous putative pathogenic variants among six unrelated sALS patients. Overall, *TP73* mutation accounted for 0.60% of Chinese sALS patients, which was similar with previously reported in Russell’s research (0.60% & 0.82%), both implying that it is an uncommon genetic determination in ALS population worldwide. Interestingly, we discovered that exon 14 of the *TP73* gene appeared to be a mutant hotspot in the Chinese ALS cohort, unlike the earlier studies where the mutated sites were relatively clustered in exons eight, nine, ten, eleven and twelve (Figure 1A; Russell et al., 2021; Dilliott et al., 2022). To clarify whether exon 14 was simply more prone to variation and under less selection pressure in the Asian population, we examined the distribution of rare *TP73* variants found in HCs that met similar criteria (except for the (2) in the inclusion criteria). The nine rare variants detected in the control group were dispersed across whole coding regions of *TP73*, with just one mutant site situated in exon 14, which did not exhibit any mutant site clustering (Supplementary Table S8; Figure 1A). These data provided support to the hypothesis that exon 14 may be a hotspot mutation in the Chinese ALS population. However, there was no significant difference in the frequency of rare *TP73* variants within exon 14 between cases and controls (*p* = 0.1789). In the current study, we found four of the six (66.7%) variants at exon 14 (Figure 1A), which were all located in the C-terminal functional domains of p73 protein. Variants p.R538H and p.R543Q are in the SAM region, which is involved in hetero-oligomerization; variants p.L560P and R579H vitiations are in the TIA region, which is critical for suppressing its own transcriptional activity. This distribution of mutant sites may be unique to Chinese ALS patients, due to the combined effect of different ethnic origins and environmental circumstances. More multicenter research with larger sample sizes is needed to validate it further. Moreover, the functional impact of these six rare, putative pathogenic variants should be studied to clarify the molecular etiology of ALS, too.

In terms of the genotype–phenotype correlations, we systematically described the clinical manifestations of these six patients with rare, putative pathogenic *TP73* variations to establish the existence of any commonalities between these individuals. Among the six carriers of *TP73* mutations, there were both pure ALS patients and ALS patient with concomitant frontotemporal dementia. Compared with the common features of Chinese ALS population, the patients only carrying rare, putative pathogenic *TP73* mutations showed a later age at onset (61.5 vs. 54.3 years), the lower disease duration (19.25 months vs. 71 months), and the higher spinal onset rate (100% vs. 76.6%) (Chen et al., 2015; Liu et al., 2021). These findings suggest that ALS patients with only rare, putative pathogenic *TP73* mutations tend to have a late onset, a rapidly progressive course, and a poorer prognosis. Next, we further analyzed the clinical characteristics of patients with ALS with multiple variants in *TP73* and other ALS-related genes. We observed an obviously earlier disease onset in these two patients when compared to ALS patients with only *KIF5A*, or *CCNF* mutations (Nicolas et al., 2018; Tian et al., 2018). Based on these data, we deduced that *TP73* also exerts disease-modifying effects in the presence of a combination with other rare variants in the known causative genes for ALS, mainly in accelerating the age at onset, rather than shortening survival time. Our current findings corroborate the previously published research that the burden of multiple rare variants advances the age at onset of ALS (Cady et al., 2015). Nevertheless, there is no clear relationship between mutation sites and clinical phenotypes.

A most recent publication by Li et al. (2022) identified 24 rare *TP73* variants among 34 sALS patients in a large Chinese ALS cohort and briefly described the clinical presentation of these patients having rare *TP73* mutation: the average age at onset was 54.32 (11.76) years, with a sex ratio of 1.45:1. There was no data on disease progression or survival available. Our findings suggest that patients with ALS with only rare, putative pathogenic *TP73* mutations tend to have a late onset, faster progression, and worse prognosis. The following might be the causes of these clinical phenotypic contradictions: Patients and controls in Li, Chunyu et al’s and our studies came from different Chinese areas; The criteria for rare, pathogenic variants in Li, Chunyu et al’s study were inconsistent with those in our study; Two studies varied in the exclusion of patients with ALS with known pathogenic mutations in established ALS genes, making Li, Chunyu et al’ patients’ clinical presentation more complex and variable; However, in the present study, we tightly restricted the rarity and pathogenicity of *TP73* variations, and separated these ALS patients with *TP73* mutation into two subgroups for further clinical phenotype analysis (one subgroup with only *TP73* mutations, the other with multiple mutations).

Herein, we also found two common *TP73* variants in the exon-intron boundaries associated with ALS, and both can lead to a reduced P73 protein expression in the human brain based on the eQTL analysis. In addition, we noted an enrichment of rare variants in the UTRs of *TP73* among our ALS patients. Increasing evidence has shown that variations in the UTRs are strongly linked to human diseases by impacting the transcription of nearby genes and protein expression level by changing the poly(A) motifs, RNA secondary structure, and RNA binding protein-binding sites (Li et al., 2021). From these findings, we suggest that these ALS-related *TP73* variants in the UTRs and exon-intron boundaries may have a haploinsufficiency effect that confers a loss-of-function phenotype, and hence impart a risk of ALS. Of course, further biological research is warranted to validate this concept.

In summary, we screened *TP73* variants in a large Chinese ALS cohort and identified six rare, candidate pathogenic mutations in six unrelated sALS patients. We provide the systematical characterization of the clinical manifestations of ALS patients carrying *TP73* mutations and investigate the phenotype–genotype associations. Our research expands the genotypic and phenotypic spectrum of *TP73* mutations in the ALS-FTD spectrum, adding to our current understanding of the characteristic clinical phenotype of ALS patients carrying rare pathogenic *TP73* variants. These results may contribute to a better grasp of the molecular mechanism of ALS. More research with larger sample size and robust functional studies are warranted to elucidate the contribution and potential molecular mechanisms of *TP73* mutations in the ALS-FTD spectrum.

## Data availability statement

The original contributions presented in the study are included in the article/Supplementary material, further inquiries can be directed to the corresponding author.

## Ethics statement

The studies involving human participants were reviewed and approved by the Ethics Committee of Xiangya Hospital of Central South University in China. The patients/participants provided their written informed consent to participate in this study.

## Author contributions

XT performed the majority of the analyses and wrote the manuscript. YY and ZL contributed to the bioinformatic analyses. YB, LT, and QZ contribute to the collection of detailed clinical data. BJ, JG, LS, and HJ contributed to the clinical part of the project. BT contributed to the supervision of the analyses. JW designed the study and wrote the manuscript. All authors contributed to the article and approved the submitted version.

## Funding

This work was supported by the Science and Technology Innovation 2030 (STI2030-Major Projects:2021ZD0201803); National Key R&D Program of China (No. 2021YFA0805202 and 2018YFC1312003); the Program of the National Natural Science Foundation of China (#82171431, 81671120 and 81300981); the National Key Research and Development Program of China (#2018YFC1312003); the Natural Science Fund for Distinguished Young Scholars of Hunan Province, China (#2020JJ2057); the Project Program of National Clinical Research Center for Geriatric Disorders at Xiangya Hospital (#2020LNJJ13).

## Conflict of interest

The authors declare that the research was conducted in the absence of any commercial or financial relationships that could be construed as a potential conflict of interest.

## Publisher’s note

All claims expressed in this article are solely those of the authors and do not necessarily represent those of their affiliated organizations, or those of the publisher, the editors and the reviewers. Any product that may be evaluated in this article, or claim that may be made by its manufacturer, is not guaranteed or endorsed by the publisher.
