# Supplementary material for: Genetic and clinical analysis of TP73 gene in amyotrophic lateral sclerosis patients from Chinese mainland
Source: Front Aging Neurosci. 2023 Feb 9;15:1114022. doi: 10.3389/fnagi.2023.1114022 (PMC9947132; doi:10.3389/fnagi.2023.1114022)
Supplement: Supplementary file 1 [file Data_Sheet_1.pdf]

Supplementary Material

Genetic and clinical analysis of *TP73* gene in amyotrophic lateral sclerosis patients from Chinese mainland

Xuxiong Tang[1], Yanchun Yuan MD[1], Zhen Liu MD[1], Yue Bu[1], Linxin Tang[1], Qianqian Zhao[1], Bin Jiao[1.3.4.5.6], Jifeng Guo[1.3.4.5.6], Lu Shen MD[1.3.4.5.6], Hong Jiang MD[1.2.3.4.5], Beisha Tang MD[1.3.4.5.6], Junling Wang MD[1.3.4.5.6]*

*** Correspondence:** Corresponding Author: junling.wang@csu.edu.cn

Supplementary Table 1. Demographic characteristics of ALS patients and healthy controls

	ALS group	healthy controls group
Patients with ALS, n	985	1224
Family history, n	69	NA
Male, n (%)	685(69.54)	577(47.14)
Age at onset, y	54.15 ± 10.76	NA
Age at sampling, y	56.21 ± 11.39	68.47 ± 6.30
Limb onset, n (%)	740(75.13)	NA
Bulbar onset, n (%)	191(19.39)	NA
Limb & bulbar onset, n (%)	54(5.48)	NA

Abbreviations: ALS = amyotrophic lateral sclerosis; NA = data not available.

Supplementary Table 2. Details of rare, putative pathogenic variants in *TP73* identified in patients with ALS in this study

Numbers. of patients with ALS	[a]Chromosome position	dbSNP	Exon	cDNA alteration	Amino acid change	Mutation type	Allele frequency gnomAD_EAS	Allele frequency ESP6500s_EAS	Allele frequency ExAC_EAS	Allele frequency 1000G_EAS	Functional predictions (pathogenic/total)
1	chr1:3624113	-	4	c.187G>A	p.A63T	Missense	-	-	-	-	5/11
1	chr1:3646593	rs146810167	11	c.1226C>T	p.P409L	Missense	-	-	-	-	8/11
1	chr1:3649345	rs745542298	14	c.1613G>A	p.R538H	Missense	-	-	-	-	11/11
1	chr1:3649360	rs779902863	14	c.1628G>A	p.R543Q	Missense	8.56E-04	-	8.00E-04	-	11/11
1	chr1:3649411	rs139568604	14	c.1679T>C	p.L560P	Missense	2.00E-04	1.00E-04	1.00E-04	-	10/11
1	chr1:3649468	rs376429700	14	c.1736G>A	p.R579H	Missense	-	1.00E-04	-	-	9/11

Key: -, not present or zero; cDNA, complementary deoxyribonucleic acid; dbSNP, database of single nucleotide; gnomAD, genomes aggregation database; ESP6500s, the NHLBI Exome Sequencing Project; ExAC, the Exome Aggregation Consortium; 1000G, the 1000 Genome Project; [a] Position on Genome Reference Consortium human genome build 37 (GRCh37); transcript NM_005427 has been used for TP73 variants nomenclature.

Supplementary Table 3. Function prediction of the identified rare, putative pathogenic variants in *TP73* by in-silico tools

Variants	SIFT	Polyphen 2 HDIV	Polyphen 2 HVAR	LRT	Mutation taster	Mutation assessor	FATHMM	MetaSVM	MetaLR	MCAP	CADD
c.187G>A (p.A63T)	Tolerable (0.435)	Benign (0.376)	Benign (0.142)	Unknown (0)	Disease Causing (1)	Low (1.1)	Damaging (-5.54)	Damaging (0.605)	Damaging (0.888)	Damaging (0.717)	Tolerable (10.66)
c.1226C>T (p.P409L)	Tolerable (0.108)	Possibly damaging (0.581)	Benign (0.086)	Deleterious (0)	Disease Causing (1)	Medium (2.455)	Damaging (-5.73)	Damaging (1.021)	Damaging (0.936)	Damaging (0.714)	Tolerable (17.44)
c.1613G>A (p.R538H)	Damaging (0.002)	Probably damaging (0.999)	Probably damaging (0.965)	Deleterious (0)	Disease Causing (1)	Medium (2.295)	Damaging (-1.53)	Damaging (1.072)	Damaging (0.973)	Damaging (0.649)	Damaging (33)
c.1628G>A (p.R543Q)	Damaging (0.007)	Probably damaging (1)	Probably damaging (0.997)	Deleterious (0)	Disease Causing (1)	Medium (2.08)	Damaging (-1.8)	Damaging (1.095)	Damaging (0.975)	Damaging (0.645)	Damaging (34)
c.1679T>C (p.L560P)	Damaging (0.001)	Probably damaging (0.993)	Possibly damaging (0.882)	Deleterious (0)	Disease Causing (1)	Low (1.585)	Damaging (-6.54)	Damaging (1.057)	Damaging (0.977)	Damaging (0.905)	Damaging (24.6)
c.1736G>A (p.R579H)	Tolerable (0.154)	Probably damaging	Probably damaging	Deleterious	Disease Causing	Low (0.975)	Damaging (-2.98)	Damaging (0.681)	Damaging (0.783)	Damaging (0.339)	Damaging (26.9)

(1)	(0.999)	(0)	(1)

SIFT, Sorting Intolerant from Tolerant; PolyPhen2 HDIV, Polymorphism Phenotyping Version 2 Human Diversity; PolyPhen2 HVAR, Polymorphism Phenotyping Version 2 Human Variation; LRT, likelihood ratio test; FATHMM, functional analysis through hidden Markov models; SVM, support vector machine; LR, logistic regression; MCAP, Mendelian clinically applicable pathogenicity; CADD, combined annotation dependent depletion.

Supplementary Table 4. List of the known ALS genes included in this study

ALS2	*ANG*	*ANXA11*	*ATP13A2*	*ATXN2*	*CACNA1H*	*CCNF*	*CHCHD10*	*CHMP2B*	*C9orf72*
C19orf12	*C21orf2*	*DAO*	*DCTN1*	*DJ1*	*DNAJC7*	*ELP3*	*ERBB4*	*ERLIN1*	*EWSR1*
FIG4	*FUS*	*GARS*	*GLE1*	*GLT8D1*	*GRN*	*hnRNPA1*	*hnRNPA2B1*	*KIF5A*	*MATR3*
NEFH	*OPTN*	*PFN1*	*PRPH*	*SETX*	*SIGMAR1*	*SOD1*	*SPG11*	*SQSTM1*	*SS18L1*
SYNE1	*TAF15*	*TARDBP*	*TBK1*	*TIA1*	*TUBA4A*	*UBQLN2*	*VAPB*	*VCP*	*VRK1*

Supplementary Table 5. 36 common *TP73* variants identified in our cohort

Position	Ref	Alt	dbSNP	Frequency	N	P-value	P^{*}-value	SE
Chr1:3644349	A	G	rs2181486	0.30149389	2209	0.00199664	0.03593952	0.01683281
Chr1:3644374	A	G	rs2146657	0.29968312	2209	0.0022571	0.04062775	0.01687655
Chr1:3646214	T	C	rs1885866	0.29877773	2209	0.00575586	0.1036055	0.01684254
Chr1:3646192	G	A	rs1885867	0.29311906	2209	0.00857987	0.15443771	0.0169878
Chr1:3646291	G	A	rs1885865	0.29311906	2209	0.00857987	0.15443771	0.0169878
Chr1:3644085	G	A	rs10910017	0.24671797	2209	0.02850752	0.51313531	0.01814686
Chr1:3645835	C	A	rs2254530	0.24603893	2209	0.03338934	0.60100814	0.01820651
Chr1:3646137	C	T	rs11589885	0.24694432	2209	0.04319402	0.77749241	0.01820831
Chr1:3599514	C	T	rs57596460	0.1505206	2209	0.07127521	1.28295385	0.02108407
Chr1:3647712	C	G	rs60679866	0.01697601	2209	0.07472362	1.34502516	0.05799175
Chr1:3643927	A	G	rs2296031	0.04572205	2209	0.08335518	1.50039331	0.03600314
Chr1:3599750	TG	T	rs796864922	0.15119964	2209	0.09385085	1.68931526	0.02102851
Chr1:3644669	C	T	rs2236367	0.04594839	2209	0.10051872	1.80933687	0.03593396
Chr1:3644858	G	C	rs2236365	0.04594839	2209	0.10051872	1.80933687	0.03593396
Chr1:3607520	G	A	rs3765730	0.3809416	2209	0.12143417	2.18581497	0.01544944
Chr1:3649562	G	A	rs9662633	0.24060661	2209	0.19127173	3.44289114	0.01867316
Chr1:3649403	G	A	rs61735051	0.24083296	2209	0.20527194	3.69489494	0.01867745
Chr1:3607355	G	A	rs3765729	0.02716161	2209	0.21506371	3.87114674	0.04589154
Chr1:3649802	G	C	rs370630845	0.01425985	2209	0.22895067	4.12111204	0.06204869

Chr1:3649794	A	C	rs9659687	0.2410593	2209	0.24811144	4.46600587	0.01864784
Chr1:3649805	A	G	rs9659688	0.24015392	2209	0.25362429	4.56523726	0.01866876
Chr1:3644715	C	T	rs12046074	0.24241738	2209	0.26832466	4.82984379	0.01855181
Chr1:3644754	T	C	rs12048341	0.24264373	2209	0.28573005	5.1431409	0.01854978
Chr1:3638674	C	T	rs1801174	0.48008149	2209	0.30510141	5.49182534	0.01524056
Chr1:3647962	T	C	rs1745813	0.48868266	2209	0.30567676	5.50218164	0.01550837
Chr1:3647877	T	C	rs7517037	0.2376641	2209	0.37186674	6.69360137	0.0186552
Chr1:3647419	G	A	rs41301973	0.23856949	2209	0.39918478	7.18532599	0.01865499
Chr1:3647944	C	A	rs12569205	0.23675871	2209	0.41471616	7.46489088	0.01868184
Chr1:3598899	C	T	rs5031052	0.01561793	2209	0.45415556	8.1748001	0.05950327
Chr1:3645844	G	A	rs2096224	0.48755093	2209	0.47273569	8.5092424	0.01555619
Chr1:3638834	C	T	rs12737840	0.01041195	2209	0.69104807	12.4388652	0.07339325
Chr1:3644805	A	G	rs2236366	0.28927116	2209	0.79752905	14.3555228	0.0173858
Chr1:3607606	C	A	rs3819957	0.03440471	2209	0.82591128	14.866403	0.04140877
Chr1:3638822	G	A	rs370902308	0.02195564	2209	0.85129702	15.3233463	0.04982306
Chr1:3598910	C	T	rs1801173	0.22159348	2209	0.94617928	17.0312271	0.01781955
Chr1:3598900	G	A	rs2273953	0.22114079	2209	0.96812498	17.4262497	0.01783139

Abbreviations: Chr: chromosome; Ref: reference allele; Alt: alternate allele; dbSNP: dbSNP137 (https://www.ncbi.nlm.nih.gov/snp/); N: number of subjects in this study; P^*-value: P-value after the Bonferroni correction; SE: standard error

Supplementary Table 6. Block statistics of the 24 common variants

	CHR	Genomic region of block (bp)	Total block length (kb)	No.of SNPs in blocks(n)	SNPs
Block 1	1	3598900 - 3598910	0.011	2	rs2273953 rs1801173
Block 2	1	3599514 - 3599750	0.237	2	rs57596460 rs796864922
Block 3	1	3638674 - 3644085	5.412	2	rs1801174 rs10910017
Block 4	1	3644349 - 3644374	0.026	2	rs2181486 rs2146657
Block 5	1	3644715 - 3645835	1.121	4	rs12046074 rs12048341 rs2236366 rs2254530
Block 6	1	3646137 - 3649805	3.669	12	rs11589885 rs1885867 rs1885866 rs1885865 rs41301973 rs7517037 rs12569205 rs1745813 rs61735051 rs9662633 rs9659687 rs9659688

Abbreviations: Chr: chromosome; SNP: single nucleotide polymorphism; No.of SNPs in blocks(n): the number of SNPs in the block

Supplementary Table 7. Burden analysis of rare variants across different genome regions of the *TP73* gene (the frequency of carriers <0.01 in all participants)

Genome region	P-value
Coding regions	1
Untranslated regions	0.0461
Intron-exon boundaries	0.5710

Supplementary Table 8. nine rare *TP73* variants detected in the control group in our study

Numbers. of healthy controls	[a]Choromosome position	dbSNP	Exon	cDNA alteration	Amino acid change	Mutation type	Allele frequency gnomAD_EAS	Allele frequency ExAC_EAS	Functional predictions (pathogenic/total)
1	chr1:3598961	rs192866394	2	c.32G>T	p.G11V	Missense	0	0	5/11
1	chr1:3624261	rs377337435	4	c.335C>T	p.A112V	Missense	5.80E-05	0.0001	10/11
1	chr1:3644212	rs528016587	8	c.863G>A	p.R288Q	Missense	6.28E-05	0	9/11
2	chr1:3644710	rs746096915	9	c.1003C>A	p.P335T	Missense	5.81E-05	0.0001	6/11
1	chr1:3644739	rs755720136	9	c.1032_1034del	p.344_345del	nonframeshift deletion	0.0001	0.0002	-
1	chr1:3645909	-	10	c.1093T>C	p.F365L	Missense	-	-	10/11
1	chr1:3648047	-	13	c.1505G>A	p.C502Y	Missense	-	-	10/11
1	chr1:3648116	rs770506799	13	c.1574T>C	p.I525T	Missense	5.80E-05	0	5/11
1	chr1:3649446	rs775611773	14	c.1714G>A	p.G572S	Missense	0	0	9/11

Key: -, not present or zero; cDNA, complementary deoxyribonucleic acid; dbSNP, database of single nucleotide; gnomAD, genomes aggregation database; ESP6500s, the NHLBI Exome Sequencing Project; ExAC, the Exome Aggregation Consortium; 1000G, the 1000 Genome Project; [a] Position on Genome Reference Consortium human genome build 37 (GRCh37); transcript NM_005427 has been used for *TP73* variants nomenclature.

Supplementary Figure 1. Brian MRI of Patient S0423 and S4096

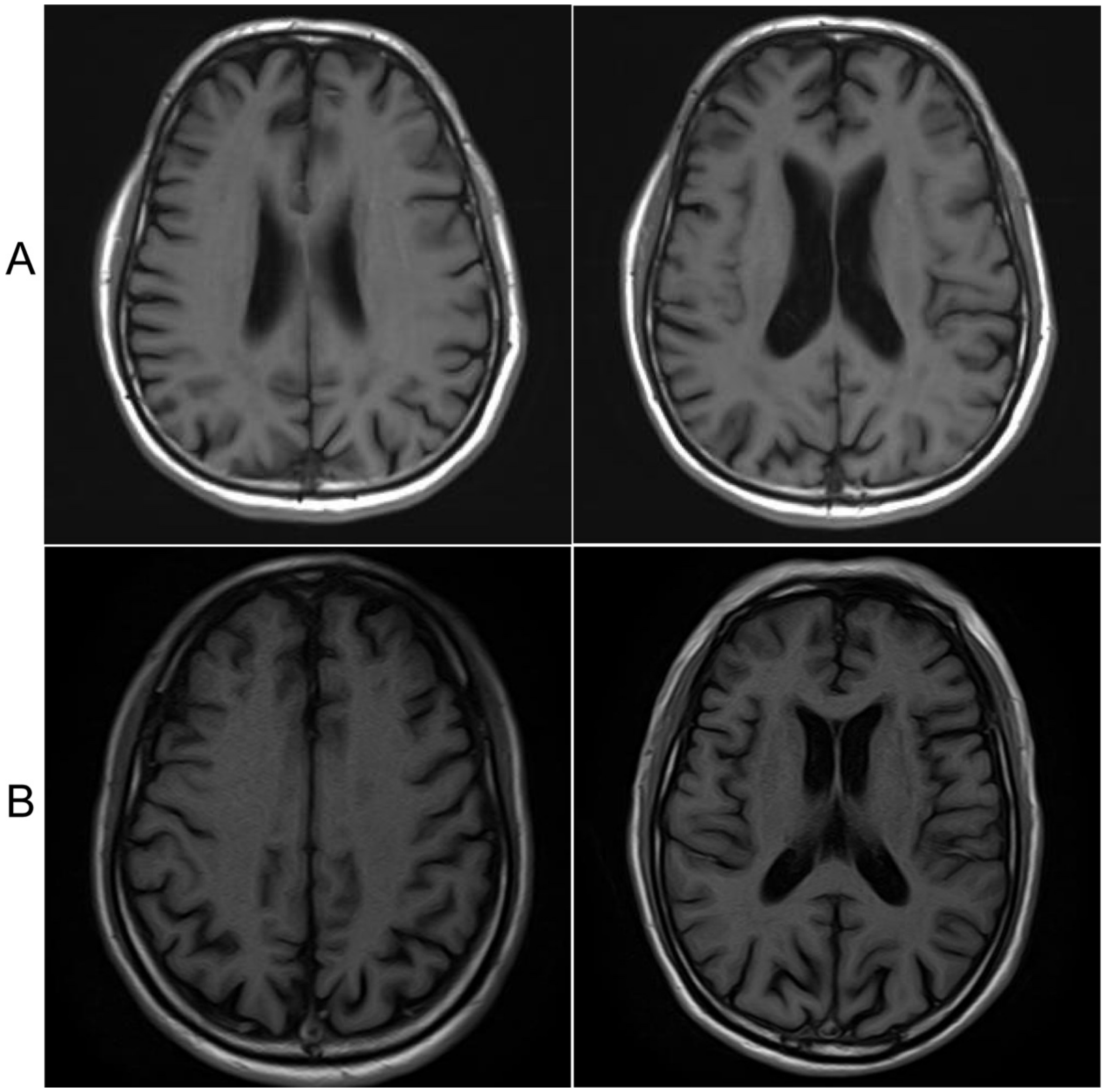

A. Brain MRI of patient A0423

B. Brain MRI of patient S4096
